# Cigarette smoking prevalence in US counties: 1996-2012

**DOI:** 10.1186/1478-7954-12-5

**Published:** 2014-03-24

**Authors:** Laura Dwyer-Lindgren, Ali H Mokdad, Tanja Srebotnjak, Abraham D Flaxman, Gillian M Hansen, Christopher JL Murray

**Affiliations:** 1 Institute for Health Metrics and Evaluation, University of Washington, 2301 5th Ave, Suite 600, Seattle, WA 98121, USA; 2 Ecologic Institute, Pfalzburger Str. 43/44, Berlin 10717, Germany

**Keywords:** Smoking, Tobacco, Disparities, BRFSS, Geographic patterns, Small area estimation

## Abstract

**Background:**

Cigarette smoking is a leading risk factor for morbidity and premature mortality in the United States, yet information about smoking prevalence and trends is not routinely available below the state level, impeding local-level action.

**Methods:**

We used data on 4.7 million adults age 18 and older from the Behavioral Risk Factor Surveillance System (BRFSS) from 1996 to 2012. We derived cigarette smoking status from self-reported data in the BRFSS and applied validated small area estimation methods to generate estimates of current total cigarette smoking prevalence and current daily cigarette smoking prevalence for 3,127 counties and county equivalents annually from 1996 to 2012. We applied a novel method to correct for bias resulting from the exclusion of the wireless-only population in the BRFSS prior to 2011.

**Results:**

Total cigarette smoking prevalence varies dramatically between counties, even within states, ranging from 9.9% to 41.5% for males and from 5.8% to 40.8% for females in 2012. Counties in the South, particularly in Kentucky, Tennessee, and West Virginia, as well as those with large Native American populations, have the highest rates of total cigarette smoking, while counties in Utah and other Western states have the lowest. Overall, total cigarette smoking prevalence declined between 1996 and 2012 with a median decline across counties of 0.9% per year for males and 0.6% per year for females, and rates of decline for males and females in some counties exceeded 3% per year. Statistically significant declines were concentrated in a relatively small number of counties, however, and more counties saw statistically significant declines in male cigarette smoking prevalence (39.8% of counties) than in female cigarette smoking prevalence (16.2%). Rates of decline varied by income level: counties in the top quintile in terms of income experienced noticeably faster declines than those in the bottom quintile.

**Conclusions:**

County-level estimates of cigarette smoking prevalence provide a unique opportunity to assess where prevalence remains high and where progress has been slow. These estimates provide the data needed to better develop and implement strategies at a local and at a state level to further reduce the burden imposed by cigarette smoking.

## Introduction

Tobacco consumption is a leading risk factor for morbidity and premature mortality in the United States (US) [1-4]. While cigarette smoking prevalence has been declining at the national level, there is substantial variation across states within the US and reason to believe that even more variation may exist at local levels, such as counties [5-7].

Evidence-based and cost-effective strategies for reducing the burden of tobacco are available [8,9]. States have differed in their uptake of these strategies, however, and have also seen varying degrees of success in reducing the prevalence of cigarette smoking and the associated disease burden, deaths, and costs to the health care system [10]. In the US, local jurisdictions have the ability to implement their own tobacco control policies and programs. Further, state-level policies may not be implemented or enforced evenly across all jurisdictions. Consequently it is essential that local estimates of current cigarette smoking prevalence are available for identifying areas that need further attention, for tracking progress, and for evaluating the effectiveness of control measures.

The US Centers for Disease Control and Prevention (CDC) routinely reports current cigarette smoking prevalence at the state level using data from the Behavioral Risk Factor Surveillance System (BRFSS) [10,11]. More local assessments have been published for some, but not all, jurisdictions [12-15]. The County Health Rankings & Roadmaps program [14] incorporates county-level estimates of current cigarette smoking prevalence into their annual rankings of counties based on selected health outcomes and health behaviors. These estimates use BRFSS data but are averages over long time periods and do not provide the means to look at estimates for specific years or trends over time. The National Cancer Institute has produced estimates of current cigarette smoking prevalence for health service areas and counties for two periods, 1997-1999 and 2000-2003, but these estimates have not been updated to include data from the last decade [16]. Indeed, to our knowledge there has been no recent, comprehensive assessment of trends in current cigarette smoking prevalence at the county level using a consistent statistical methodology applied to all counties. In this study, we develop county-level measurements of cigarette smoking prevalence for all counties in the United States annually from 1996 to 2012.

## Methods

### Data

We utilize county-level data on cigarette smoking from the BRFSS. The BRFSS is a telephone survey in which trained interviewers in each state collect data on a large number of health-related behaviors and conditions for the noninstitutionalized adult population. The BRFSS is operated by state health departments in collaboration with the CDC, and all states implement the same core questionnaire. Beginning in 2011, the BRFSS incorporated cell phones into the sampling frame in addition to landlines in order to capture the growing segment of the population that only receives calls on a cell phone. Details on BRFSS methodology are available elsewhere [17,18], and questionnaires and data are available at http://www.cdc.gov/brfss. Alaska conducts a supplemental BRFSS using the same methodology as the standard BRFSS [19]; data from the Alaska supplemental BRFSS on cigarette smoking were included from 2004 to 2012 in addition to data from the standard BRFSS.

Cigarette smoking status was assessed using two questions from the BRFSS [20]. Respondents were first asked, “Have you smoked at least 100 cigarettes in your entire life?” If a respondent answered yes, he or she was then asked, “Do you now smoke cigarettes every day, some days or not at all?” We used the responses to these two questions to classify respondents into three groups: nonsmokers (those who answer “no” to the first question or “not at all” to the second question), nondaily current smokers (those who answer “some days” to the second question), and daily current smokers (those who answer “every day” to the second question). We estimate the prevalence of current total cigarette smoking (both nondaily and daily combined; hereafter referred to as “total cigarette smoking prevalence”) as well as the prevalence of current daily cigarette smoking only (hereafter referred to as “daily cigarette smoking prevalence”).

### Small area estimation models

We applied previously described small area models to estimate the prevalence of cigarette smoking for US counties [21-23]. In brief, we constructed a family of logistic hierarchical mixed effects regression models for each outcome, stratified by sex. These models incorporate spatial and temporal smoothing and a series of county- and state-level covariates to improve predictions for all counties, including those with limited data available in a given year from the BRFSS. More details on the regression models and the county- and state-level data sources incorporated in the models can be found in Additional files 1 and 2. These models allowed us to generate annual estimates of total and daily cigarette smoking prevalence for male and female adults (age 18 and older) in all US counties and county equivalents. All estimates were age-standardized following the age structure of the 2000 census [24]. The uncertainty of the prevalence estimates was assessed using simulation methods [25].

### Model validation and performance assessment

We also used previously developed validation methods [21-23] to select the best-performing model among a number of different plausible models. Our approach was as follows: for each sex we selected counties with at least 900 survey respondents between 2006 and 2010 (the “validation set”); 900 was selected in previous investigations based on simulation studies as the number that generated sufficiently precise estimates for a wide range of outcomes. Using the pooled data for this time period, we calculated a “gold standard” estimate of cigarette smoking prevalence for each county in the validation set. We then created new datasets by generating random samples from counties in the validation set of size 10, 50, and 100 respondents per year. Next, we used these “sampled-down” datasets to fit each model and compared the resulting prevalence estimates for counties in the validation set with the gold standard. We measured model performance using the concordance correlation coefficient, which is a measure of the agreement between the model predictions and the gold standard, and the root mean squared error, a measure of the magnitude of the deviation between the model predictions and the gold standard, expressed in the same units as the predictions.

### Bias correction for wireless-only households

In 2011 cell phones were introduced into the BRFSS sampling frame in order to capture the growing share of the adult population that is “wireless-only” (36.5% as of the second half of 2012 [26]) and cannot be reached by landline. Previous research has suggested that cigarette smoking prevalence is different among wireless-only respondents and respondents who can be reached by landline, and that omitting wireless-only respondents from a survey will bias estimates of cigarette smoking prevalence, most likely leading to underestimates [27,28]. We used two complementary approaches to address the omission of wireless-only respondents from the BRFSS sampling frame prior to 2011. First, we incorporated a number of demographic characteristics—race, marital status, and educational achievement—that are related both to phone ownership and to cigarette smoking prevalence into the small area models. This allowed us to adjust our modeled estimates for each county to match the observed distribution of the population by these characteristics and to account for differences in the cigarette smoking prevalence between the wireless-only population and the general population that are due to differences in these factors. However, after making this adjustment, the prevalence estimates derived from the 2011 sample were higher than those derived from the 2010 sample, a marked and unlikely departure from trends observed in the recent past. Indeed, this suggested that differences in race, marital status, and education alone do not explain all of the difference in cigarette smoking prevalence between wireless-only respondents and the rest of the population. To address this bias, we fit two separate small area models: the first to data from respondents with landlines in all years (1996-2012), and the second to data from all respondents, including wireless-only respondents, in 2011 and 2012. In the second model, we included phone usage category (landline-only, dual, and wireless-only) to adjust estimates for the observed phone usage characteristics of the county. We compared the estimates for 2011 from the combined sample (the second model) to the estimates for the landline sample (the first model) to derive county-level measures of the bias introduced by not including wireless-only respondents in 2011. We assumed that this bias has increased linearly with time from no bias in the year 2000 (when relatively few adults were wireless-only) [29-31] to the level measured in 2011 and used this assumption to calculate corrected estimates in each year from 2001 to 2010. Estimates for 1996 to 2000 were based on the first model, without correction, while estimates for 2011 and 2012 were based on the second model.

### Unit of analysis

Our unit of analysis was counties or county equivalents (e.g., parishes, census enumeration areas, boroughs, and independent cities). As of 2012 there were 3,143 counties and county equivalents. To account for changes over the study period, we merged some counties to get consistent areas, for a total of 3,127 counties. There were 4,738,256 respondents age 18 and over in the BRFSS from 1996 to 2012 who had complete data for all variables of interest. In 2012 the combined response rate for cell and landline ranged from 27.7% to 60.4% with a median of 45.2% among the states. This response rate takes into account the likely number of eligible respondents among phone numbers for which eligibility could not be determined [32]. All analyses were carried out in R version 3.0.2 [33].

## Results

### Model validation and performance

The concordance correlation for the selected model for male total cigarette smoking prevalence was 0.78, 0.83, and 0.87 at sample sizes 10, 50, and 100, respectively, compared to 0.90 when all data were included (i.e., “in sample”). For women, the corresponding figures are 0.78, 0.85, 0.88, and 0.91. The root mean squared error for the selected model for male cigarette smoking prevalence was 2.7, 2.5, 2.2, and 1.9 for sample sizes 10, 50, 100, and in sample, respectively, while for women the root mean squared error was almost identical at 2.8, 2.4, 2.2, and 1.9 for the same sample sizes. Performance of the selected model for male and female daily cigarette smoking was similar to that for total cigarette smoking.

### Bias correction for wireless-only households

We compared model predictions for 2011 that incorporated respondents who could only be reached by cell phone with model predictions that did not incorporate these respondents in order to derive a correction for earlier years in which the wireless-only population was excluded. In 2011, the median difference in total cigarette smoking prevalence between modeled estimates with and without wireless-only respondents included was 1.21 percentage points for men and 1.55 percentage points for women. In 2010, the last year without cell phones, where bias due to their exclusion is expected to be greatest, we corrected 57.3% and 75.4% of counties for males and females, respectively, upward by at least one percentage point and 4.7% and 16.3% of counties for males and females, respectively, upward by at least two percentage points for total cigarette smoking prevalence.

### National total cigarette smoking prevalence

**Figure 1:**
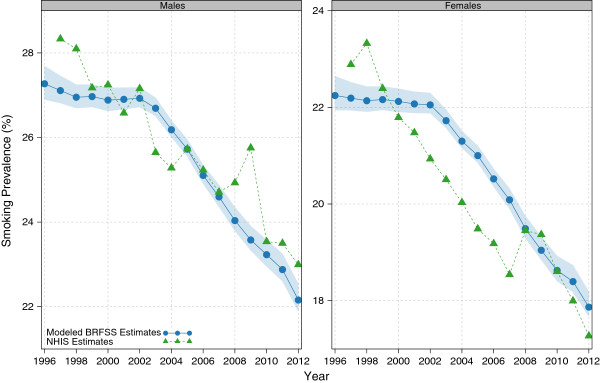
National age-standardized total cigarette smoking prevalence, 1996-2012.

Figure 1 shows the national estimates for age-standardized total cigarette smoking prevalence derived from our models. Total cigarette smoking prevalence has declined for males by 1.3% (95% uncertainty interval: 1.2%-1.4%) per year, from 27.3% (26.9%-27.7%) to 22.2% (21.9%-22.5%), and for females by 1.4% (1.2%-1.5%) per year, from 22.2% (21.9%-22.6%) to 17.9% (17.7%-18.2%). Most of this decline took place from 2002 onwards; trends from 1996 to 2002 are relatively flat.

For comparison, direct (nonmodeled) estimates from the National Health Interview Survey (NHIS) [34], a nationally representative household survey, are also plotted. These estimates have been reweighted to account for the distribution of the population by race, marital status, and educational attainment and then age-standardized; this is for consistency with the modeled BRFSS estimates. Estimates from the NHIS for total cigarette smoking confirm the declines observed in the modeled estimates based on BRFSS data. Further, while estimates from NHIS vary noticeably from year to year, on the whole the level of total cigarette smoking suggested by the NHIS is consistent with that from our models based on BRFSS data.

### County-level total cigarette smoking prevalence

**Figure 2:**
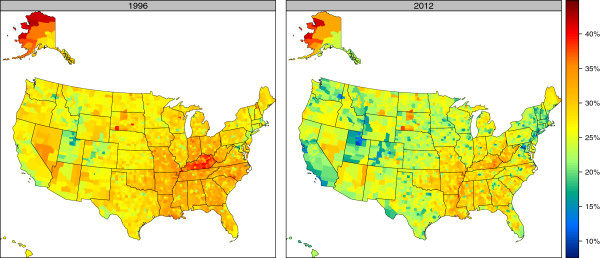
Age-standardized total cigarette smoking prevalence, males, 1996 and 2012.

**Figure 3:**
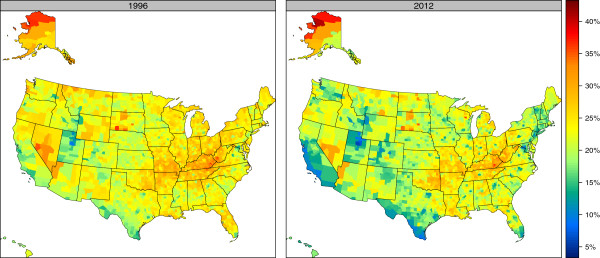
Age-standardized total cigarette smoking prevalence, females, 1996 and 2012.

Figures 2 and 3 show the age-standardized total cigarette smoking prevalence for males and females, respectively, in 1996 and 2012. (Estimates for the top and bottom 10 counties in 2012 are presented in Tables 1 and 2 for males and females, respectively, and estimates for all counties in all years are presented in Additional file 3.) For males, regions with high levels of cigarette smoking are observed in the South and parts of the Midwest, particularly around Kentucky. High levels are also observed in parts of Alaska, South Dakota, Nevada, and Arizona. Regions of noticeably low cigarette smoking among males are observed in Utah, Colorado, Wyoming, California, Washington, and parts of New England. For females, the highest levels of cigarette smoking are concentrated in Kentucky, West Virginia, Tennessee, Missouri, Oklahoma, Arkansas, and Louisiana; this pattern is somewhat different from the pattern among males where a much larger portion of the South experienced elevated cigarette smoking rates. Higher levels for females are also observed in parts of Alaska, Nevada, Arizona, North Dakota, and South Dakota, while the lowest levels are seen in Utah, Colorado, Wyoming, California, and along the Mexico-Texas border.

**Table 1:** Top- and bottom-ranked counties for male total cigarette smoking prevalence, 2012

Rank*	**County**	Age-standardized total cigarette smoking prevalence (%)*
1 (1, 6)	Falls Church City, VA	9.9 (8.1, 12.0)
2 (1, 4)	Utah County, UT	9.9 (8.8, 11.3)
3 (2, 17)	Davis County, UT	11.7 (10.2, 13.6)
4 (2, 21)	Wasatch County, UT	11.8 (9.8, 14.0)
5 (2, 23)	Arlington County, VA	11.8 (9.8, 14.2)
6 (3, 32)	Summit County, UT	12.5 (10.6, 14.7)
7 (3, 37)	Howard County, MD	12.7 (10.7, 15.1)
8 (3, 46)	Whitman County, WA	12.8 (10.4, 15.4)
9 (3, 38)	Cache County, UT	12.8 (10.8, 15.0)
10 (3, 49)	Loudoun County, VA	13.1 (10.9, 15.6)
3,118 (2,866, 3,127)	Issaquena County, MS	36.8 (31.9, 42.2)
3,119 (2,809, 3,127)	East Carroll Parish, LA	37.0 (31.6, 42.4)
3,120 (2,898, 3,126)	Clay County, KY	37.2 (32.3, 42.2)
3,121 (2,859, 3,127)	Lee County, KY	37.4 (31.9, 42.2)
3,122 (2,946, 3,126)	Bethel Census Area, AK	37.5 (33.1, 42.3)
3,123 (2,875, 3,127)	Sioux County, ND	37.7 (32.1, 43.6)
3,124 (2,879, 3,127)	Shannon County, SD	37.9 (32.2, 44.1)
3,125 (2,967, 3,126)	Nome Census Area, AK	38.1 (33.1, 42.8)
3,126 (3,082, 3,127)	Wade Hampton Census Area, AK	41.2 (35.9, 46.8)
3,127 (3,089, 3,127)	Northwest Arctic Borough, AK	41.5 (35.9, 46.8)

*Numbers in parentheses are 95% uncertainty intervals.

**Table 2:** Top- and bottom-ranked counties for female total cigarette smoking prevalence, 2012

Rank*	**County**	Age-standardized total cigarette smoking prevalence (%)*
1 (1, 2)	Utah County, UT	5.8 (4.9, 6.8)
2 (1, 8)	Wasatch County, UT	7.1 (5.7, 8.8)
3 (2, 21)	Davis County, UT	8.3 (7.1, 9.9)
4 (2, 33)	Hidalgo County, TX	8.6 (6.9, 10.8)
5 (2, 37)	San Mateo County, CA	8.7 (6.9, 10.8)
6 (2, 42)	Cameron County, TX	8.8 (6.9, 11.3)
7 (2, 37)	Summit County, UT	8.9 (7.2, 10.8)
8 (3, 36)	Santa Clara County, CA	9.0 (7.4, 10.8)
9 (3, 41)	Cache County, UT	9.1 (7.4, 11.1)
10 (7, 32)	Los Angeles County, CA	9.6 (8.6, 10.6)
3,118 (2,844, 3,126)	Elliott County, KY	34.0 (28.2, 40.7)
3,119 (2,791, 3,126)	Shannon County, SD	34.1 (27.6, 40.9)
3,120 (2,973, 3,126)	Knox County, KY	34.7 (29.8, 39.8)
3,121 (2,924, 3,127)	Buffalo County, SD	35.4 (29.2, 42.2)
3,122 (2,997, 3,126)	Nome Census Area, AK	35.8 (30.2, 41.1)
3,123 (3,002, 3,127)	Wade Hampton Census Area, AK	36.2 (30.4, 42.5)
3,124 (3,023, 3,127)	Clay County, KY	36.2 (30.8, 41.9)
3,125 (3,014, 3,127)	Menominee County, WI	36.5 (30.6, 42.5)
3,126 (3,067, 3,127)	North Slope Borough, AK	37.5 (32.2, 43.0)
3,127 (3,110, 3,127)	Northwest Arctic Borough, AK	40.8 (34.8, 46.8)

*Numbers in parentheses are 95% uncertainty intervals.

In 1996, the lowest total cigarette smoking prevalence for males was observed in Utah County, UT (15.5% [13.2%-17.6%]), while the highest was found in Northwest Arctic Borough, AK (42.6% [37.0%-48.5%]), a difference of 27.1 percentage points. In 2012, Falls Church City, VA had the lowest prevalence at 9.9% (8.1%-12.0%), while the highest prevalence was still found in Northwest Arctic Borough, AK at 41.5% (35.9%-46.8%), a 31.7 percentage point difference. For females, the lowest prevalence in 1996 was found in Utah County, UT at 9.0% (7.2%-10.8%), which is 27.8 percentage points lower than the highest-observed prevalence that year in Perry County, KY at 36.8% (31.7%-42.1%). In 2012, female cigarette smoking prevalence was still lowest in Utah County, UT (5.8% [4.9%-6.8%]), which was 35.1 percentage points lower than the highest prevalence in that year, in Northwest Arctic Borough, AK (40.8% [34.8%-46.8%]).

Even within a single state there is often substantial variation among counties. The median gap between highest and lowest cigarette smoking prevalence among counties within the same state in 2012 was 14.7 percentage points for males and 13.6 percentage points for females. The largest gap for males in 2012 was observed in Virginia, where there was a 23.6 percentage point gap in cigarette smoking prevalence for men between Sussex County (33.5% [28.6%-38.7%]) and Falls Church City (9.9% [8.1%-12.0%]). For females, the largest gap in 2012 was observed in Alaska, where there was a 25.4 percentage point gap for women between Northwest Arctic Borough (40.8% [34.8%-46.8%]) and Haines Borough (15.4% [12.3%-18.8%]).

**Figure 4:**
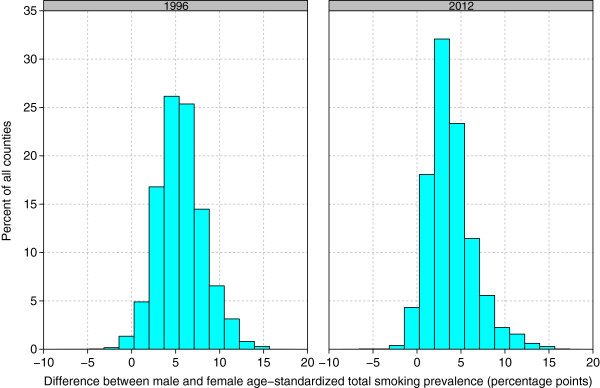
Difference between male and female age-standardized total cigarette smoking prevalence, 1996 and 2012.

In the vast majority of counties, males smoked cigarettes at higher rates than females (Figure 4): in 99.0% of counties in 1996 males had a higher cigarette smoking prevalence than females, while in 2012 the same was true in 96.4% of counties. The gap between male and female total cigarette smoking prevalence has changed with time, however: the median difference between male and female cigarette smoking was 5.4 percentage points in 1996 compared to 3.4 in 2012. Across all counties in 1996, the gap between male and female cigarette smoking ranged from -3.2 percentage points in Colonial Heights City, VA, to 15.3 percentage points in Jefferson County, MS. In 2012, the gap between male and female cigarette smoking ranged from -5.7 percentage points in Menominee County, WI, to 16.5 percentage points in Sunflower County, MS. The correlation between male and female cigarette smoking prevalence was 0.75 in 1996 and 0.81 in 2012.

**Figure 5:**
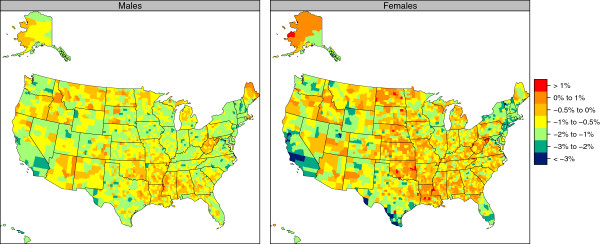
Annualized rate of change in age-standardized total cigarette smoking prevalence, 1996-2012.

**Figure 6:**
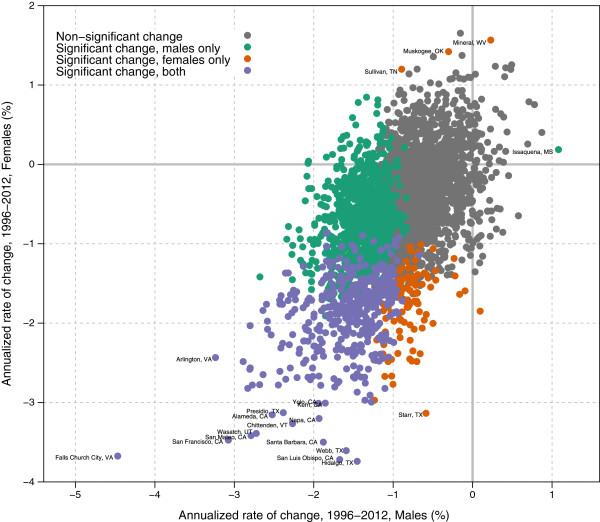
Annualized rate of change in age-standardized total cigarette smoking prevalence, females compared to males, 1996-2012.

Figures 5 and 6 show the change in age-standardized total cigarette smoking prevalence from 1996 to 2012, expressed in terms of the annualized rate of change; Tables 3 and 4 give the top and bottom 10 counties in terms of annualized rates of change. Amongst all counties, the median annualized rate of change was -0.9% for males and -0.6% for females. The greatest decline for males was 4.5% (2.6%-6.4%) per year in Falls Church City, VA, while the greatest for females was 4.1% (1.9%-6.4%) per year in Maverick County, TX. The largest increase for males was 1.1% (-0.2%-2.4%) per year in Issaquena County, MS, while the greatest increase for females was 1.7% (-0.4%-3.6%) per year in McMullen County, TX. Only 39.8% of counties for males and 16.2% of counties for females experienced statistically significant declines in cigarette smoking prevalence between 1996 and 2012, though an additional 57.3% of counties for men and 66.1% of counties for women experienced nonstatistically significant declines over this same period. Counties with statistically significant declines represent a disproportionate share of the population, however, such that 74.4% of the adult male population and 61.1% of the adult female population in 2012 lived in counties where the decline in total cigarette smoking prevalence was statistically significant. There were statistically significant increases in only one county for males and in only three counties for females. The correlation between male and female annualized rates of decline in the same county was moderate at 0.55. In most counties, males and females saw cigarette smoking prevalence move in the same direction (Figure 6), however, in 16.1% of counties males experienced declines while females experienced increases and, conversely, in 1.4% of counties females experienced declines while males experienced increases. For both males and females, the correlation between the level of cigarette smoking prevalence in 1996 and the rate of decline between 1996 and 2012 was low: 0.26 for males and 0.15 for females.

**Table 3:** Top- and bottom-ranked counties for annualized rates of change in male total cigarette smoking prevalence, 1996-2012

Rank*	**County**	Annualized rate of change in total cigarette smoking prevalence (%)*
1 (1, 87)	Falls Church City, VA	-4.5 (-6.4, -2.6)
2 (1, 555)	Arlington County, VA	-3.2 (-4.7, -1.7)
3 (1, 687)	San Francisco County, CA	-3.1 (-4.5, -1.6)
4 (2, 755)	Loudoun County, VA	-2.9 (-4.3, -1.5)
5 (2, 629)	New York County, NY	-2.8 (-4.1, -1.7)
6 (3, 745)	Orange County, CA	-2.8 (-3.9, -1.6)
7 (2, 981)	Dallas County, IA	-2.8 (-4.2, -1.3)
8 (4, 486)	Rockingham County, NH	-2.8 (-3.9, -1.8)
9 (2, 883)	San Mateo County, CA	-2.8 (-4.2, -1.4)
10 (3, 702)	Utah County, UT	-2.8 (-4.0, -1.6)
3,118 (1,795, 3,125)	Lincoln County, AR	0.4 (-0.8, 1.7)
3,119 (1,791, 3,125)	Lee County, AR	0.5 (-0.8, 1.7)
3,120 (1,625, 3,126)	Claiborne County, MS	0.5 (-0.9, 1.8)
3,121 (1,908, 3,126)	Benson County, ND	0.5 (-0.7, 1.8)
3,122 (1,784, 3,126)	Wheeler County, GA	0.6 (-0.8, 1.9)
3,123 (2,031, 3,127)	East Carroll Parish, LA	0.7 (-0.6, 2.0)
3,124 (2,321, 3,126)	Hardy County, WV	0.7 (-0.4, 1.8)
3,125 (2,177, 3,127)	Bent County, CO	0.8 (-0.5, 2.1)
3,126 (2,229, 3,127)	Meagher County, MT	0.9 (-0.5, 2.3)
3,127 (2,550, 3,127)	Issaquena County, MS	1.1 (-0.2, 2.4)

*Numbers in parentheses are 95% uncertainty intervals.

**Table 4:** Top- and bottom-ranked counties for annualized rates of change in female total cigarette smoking prevalence, 1996-2012

Rank*	**County**	Annualized rate of change in total cigarette smoking prevalence (%)*
1 (1, 441)	Maverick County, TX	-4.1 (-6.4, -1.9)
2 (1, 527)	Hidalgo County, TX	-3.7 (-5.6, -1.8)
3 (1, 596)	San Luis Obispo County, CA	-3.7 (-5.5, -1.7)
4 (1, 892)	Falls Church City, VA	-3.7 (-6.1, -1.3)
5 (1, 622)	Webb County, TX	-3.6 (-5.7, -1.5)
6 (1, 722)	Santa Barbara County, CA	-3.5 (-5.5, -1.5)
7 (2, 711)	San Francisco County, CA	-3.5 (-5.3, -1.5)
8 (1, 707)	San Mateo County, CA	-3.4 (-5.3, -1.5)
9 (1, 710)	Wasatch County, UT	-3.4 (-5.2, -1.5)
10 (7, 303)	Chittenden County, VT	-3.3 (-4.3, -2.2)
3,118 (2,208, 3,118)	Sullivan County, TN	1.2 (-0.1, 2.5)
3,119 (1,806, 3,124)	Adair County, OK	1.2 (-0.4, 2.8)
3,120 (2,002, 3,123)	Hampshire County, WV	1.2 (-0.2, 2.7)
3,121 (1,559, 3,125)	Grant County, WV	1.2 (-0.6, 3.0)
3,122 (1,837, 3,126)	Benson County, ND	1.3 (-0.4, 3.1)
3,123 (1,693, 3,126)	Bristol City, VA	1.4 (-0.5, 3.2)
3,124 (2,105, 3,126)	Allen Parish, LA	1.4 (-0.1, 3.1)
3,125 (2,148, 3,125)	Muskogee County, OK	1.4 (-0.1, 3.0)
3,126 (2,116, 3,126)	Mineral County, WV	1.6 (-0.1, 3.2)
3,127 (1,888, 3,127)	McMullen County, TX	1.7 (-0.4, 3.6)

*Numbers in parentheses are 95% uncertainty intervals.

Total cigarette smoking prevalence as well as changes in total cigarette smoking prevalence varied between counties with different mean income levels [35]. Table 5 shows the median total cigarette smoking prevalence in 1996 and 2012 and the annualized rate of decline in total cigarette smoking prevalence over this period among counties in each income quintile (defined in terms of income in 1996). In both 1996 and 2012, the median cigarette smoking prevalence decreased as mean income in 1996 increased. Moreover, the median rate of change between 1996 and 2012 was more negative for higher income quintiles than for lower income quintiles. As a consequence, more counties in higher income quintiles experienced statistically significant declines from 1996 to 2012: for males only 14.1% of counties in the bottom income quintile experienced statistically significant declines compared to 75.4% of counties in the top income quintile; for females only 4.2% of counties in the bottom income quintile experienced statistically significant declines compared to 45.2% of counties in the top income quintile.

**Table 5:** Total cigarette smoking prevalence and annualized rates of change by income quintile, 1996-2012

	**Income quintile, 1996**	Median annualized rate of change, 1996-2012 (%)*	Median age-standardized total smoking prevalence, 1996 (%)*	Median age-standardized total smoking prevalence, 2012 (%)*	**Counties with statistically significant declines between 1996 and 2012 (%)**
Males	1st quintile	-0.5 (-2.4, 1.1)	32.1 (22.8, 40.7)	29.6 (17.5, 37.9)	14.1
	2nd quintile	-0.8 (-2.3, 0.7)	30.5 (20.4, 39.5)	27.2 (18.1, 34.8)	22.7
	3rd quintile	-0.9 (-2.4, 0.4)	29.4 (19.7, 41.5)	25.6 (14.8, 41.2)	34.6
	4th quintile	-1.1 (-2.8, 0.3)	29.0 (17.5, 37.9)	24.5 (12.8, 37.5)	52.2
	5th quintile	-1.4 (-4.5, 0.0)	27.3 (15.5, 42.6)	21.8 (9.9, 41.5)	75.4
Females	1st quintile	-0.3 (-4.1, 1.4)	24.6 (14.0, 36.8)	23.6 (8.6, 36.5)	4.2
	2nd quintile	-0.4 (-2.5, 1.6)	24.8 (14.3, 35.7)	23.2 (11.7, 32.4)	5.1
	3rd quintile	-0.5 (-2.7, 1.1)	24.4 (13.6, 33.2)	22.2 (11.8, 36.2)	8.2
	4th quintile	-0.7 (-3.0, 1.7)	23.9 (11.0, 35.1)	21.4 (9.1, 31.9)	18.4
	5th quintile	-1.2 (-3.7, 0.9)	22.5 (9.0, 36.7)	18.7 (5.8, 40.8)	45.2

*Numbers in parentheses are the minimum and maximum.

### County-level daily cigarette smoking prevalence

Daily cigarette smoking prevalence is given for all counties in Additional file 4. When we examined the correlation between total and daily cigarette smoking it was very high: 0.95 across both sexes and all years combined. By definition, daily cigarette smoking is always less than total cigarette smoking prevalence, but the median difference between total and daily cigarette smoking among counties increased from 4.3 to 6.6 percentage points in males and 3.6 to 5.4 percentage points in females from 1996 to 2012. This was due to the fact that daily cigarette smoking prevalence has declined faster than total cigarette smoking prevalence: the median annualized rate of decline for daily cigarette smoking prevalence was 1.9% per year for males and 1.4% per year for females, compared to 0.9% per year and 0.6% per year for total cigarette smoking prevalence for males and females, respectively. Rates of decline in total and daily cigarette smoking over the period from 1996 to 2012 are highly correlated, however: 0.93 for both males and females. In 2012 the gap between daily and total cigarette smoking prevalence ranged from 2.7 (Utah County, UT) to 15.3 (Wade Hampton Census Area, AK) percentage points for males and from 1.4 (Utah County, UT) to 11.8 (Wade Hampton Census Area, AK) percentage points for females.

## Discussion and conclusions

Our study is the first to report on nationwide cigarette smoking prevalence and change in cigarette smoking prevalence at the county level from 1996 to 2012. Moreover, we derived these estimates using a systematic model selection and validation process. Additionally, we report on a novel method to adjust BRFSS estimates to take into account recent changes in the BRFSS methodology, which allows for analysis of trends both before and after these changes. The BRFSS has informed data users about these changes and their potential impact on the estimates and trends but has not provided a means to adjust the data. Our correction method provides a solution and allows for the seamless use of pre-2011 and post-2011 BRFSS data for research and policy analysis across the US. Our approach provides county health officials with reliable and comparable estimates of cigarette smoking prevalence for males and females in their jurisdiction and, perhaps more importantly, provides an assessment of trends in the last 17 years to assess whether a county is making as much progress as other similar counties in the US.

Our study reveals dramatic differences in cigarette smoking prevalence across the country that would not be apparent from national estimates or even state-level estimates. Indeed, within-state variation in cigarette smoking sometimes rivals variation seen in the country as a whole. State-level estimates of cigarette smoking prevalence, while useful for beginning to explore differentials within the US and indispensable for informing state-level tobacco control policies, do not provide the same level of resolution as our county estimates and hence mask important local differences in both the current level of smoking prevalence and in trends.

County-level estimates of cigarette smoking prevalence reveal pockets of high-risk populations. Our results support previous studies that have shown that cigarette smoking rates are associated with income, educational achievement, and race/ethnicity [13,36,37]. We find that very high rates of cigarette smoking appear to be a particular problem for poorer communities and those with large populations of Native Americans and Alaska natives, while lower rates of cigarette smoking are found in more affluent counties and counties with large shares of Mexican immigrants. We also find considerable geographic variation, even within states, in smoking prevalence.

Our results support previously reported findings on a decline in the prevalence of cigarette smoking in the US as a whole. However, examining trends at the county level reveals that not all counties have contributed to this decline. In reality, a relatively small proportion of counties (though representing a disproportionately large share of the population) experienced statistically significant declines over this period. We find that rates of decline in smoking prevalence at the county level for men generally exceeded those for women. We also find that declines over this period were related to income: counties in higher income brackets tended to have more rapid declines than counties in lower income brackets.

These findings illustrate the importance of county-level estimates of smoking prevalence. Progress in reducing cigarette smoking will be limited as long as so many communities are left behind. A wide range of effective tobacco control policies and programs have been developed, including excise taxes, smoke-free policies, restriction of tobacco promotion activities, quitline interventions, mass-media advertising campaigns, and policies that reduce the out-of-pocket costs related to cessation treatments [8,9,38,39]. Our estimates uniquely provide the means to assess where existing state-level policies may not be adequately enforced and where new county-level policies may be called for in lieu of or in addition to action at the state level. Further, as the tobacco industry seeks to maintain or increase sales, marketing of tobacco products is increasingly taking place at the local level [40]. Our county-level estimates provide a means of assessing and tracking the impact of such efforts.

These local, annual measurements of cigarette smoking prevalence can be an important stimulus to local public health decision-making and community engagement. Moreover, our methodology could be used to produce local estimates for other leading risk factors for the burden of disease and incorporated into a scoring system to rank counties in terms of their health performance. These kinds of county health profiles would enable local and state health officials to prioritize and target high-risk counties while spending local, state, and federal funds more wisely on prevention and treatment programs. Maintenance of health profiles over time will also allow tracking progress in confronting major risk factors. Being able to compare counties on a dollar-spent-per-point-reduction in prevalence will create positive competition and allow identification of best practices.

Recent work on global trends in daily smoking prevalence deserves mention despite the difference in definition of smoking prevalence employed (all types of tobacco, not just cigarettes as in the current study) [41]. For males, the counties with the lowest daily smoking prevalence are comparable to those countries with the lowest daily smoking prevalence globally: indeed, less than 0.5% of countries have lower male daily smoking prevalence than these counties. At the same time, counties with the highest daily smoking prevalence are comparable to countries with moderately high daily smoking prevalence globally: slightly more than one-third (36.9%) of countries have higher smoking prevalence among men than these counties. For females the comparison is quite different: 48.1% of countries have lower female daily smoking prevalence than the lowest daily smoking prevalence in any county in the US. At the same time, only 1.6% of countries have higher female daily smoking prevalence than the highest daily smoking prevalence among counties in the US.

We have used the BRFSS to develop county-level measurements of cigarette smoking prevalence, and its limitations need to be taken into account when using or interpreting our results. First, the BRFSS is a telephone survey and is subject to bias as a result of excluding the population that has no phone line. This represents a relatively small proportion of the population (less than 2% [26]), however, so the potential for bias is limited. Second, the BRFSS relies on self-reported smoking status and is therefore subject to self-reporting bias, which may vary by sex and by age. Third, while CDC makes BRFSS data available for all respondents in each survey, not all county identifiers are released: in particular, the county identifier for respondents from very small counties is typically masked. In some cases we have been able to obtain data directly from states and recover the county of residence for these respondents. However, we have not been able to do this for all respondents, particularly in recent years, and consequently are not able to make use of the entire BRFSS dataset in this analysis. Finally, the statistical models that we employ are also subject to error. While we have rigorously validated the model, this validation is internal to the dataset used for modeling—BRFSS—and cannot assess how well the model will perform in the presence of errors or biases in the BRFSS data. While estimates from the NHIS for total smoking are relatively consistent with our modeled estimates based on BRFSS data, there is still room for further research into why these two data sources are not more closely aligned. Further, while the correction method we employ for addressing the exclusion of wireless-only respondents prior to 2011 has the expected effect and brings our estimates of total smoking more closely in line with those from the NHIS, we have not been able to validate this methodology as thoroughly as we have the small area models, nor are we able to verify the assumption that bias due to exclusion of wireless-only respondents has increased linearly with time.

Over the past two decades, states and counties have introduced a number of policies and programs to address the tobacco epidemic. We find, however, that in a troublingly large number of counties there has been relatively little progress in reducing cigarette smoking prevalence. Many areas of the country are still smoking at levels found in previous decades when cigarette smoking was not yet widely recognized as a major risk factor for morbidity and premature mortality. Smoking is a leading cause of death and deserves acute attention by health and medical professionals. Public health is local, and we believe that our study provides the necessary tools to understand and measure patterns of smoking at the local level with existing data.

## Competing interests

The authors declare that they have no competing interests.

## Authors’ contributions

LD-L, AHM, TS, and CJLM developed and applied the model to estimate cigarette smoking prevalence by sex, year, and county. AHM and CJLM designed the overall study and analytical strategy. AF advised on the modeling strategy to adjust for the exclusion of cell phones. AHM and LD-L wrote the first draft. AHM, LD-L, TS, AF, GH, and CJLM revised the paper. All authors have read and approved the final manuscript.

## Supplementary Material

Additional file 1 Detailed description of small area models and data sources. Click here for file

Additional file 2 BRFSS sample size by county and year, 1996-2012. Click here for file

Additional file 3 Age-standardized total cigarette smoking prevalence, all counties, 1996-2012. Click here for file

Additional file 4 Age-standardized daily cigarette smoking prevalence, all counties, 1996-2012. Click here for file
