# Supplementary material for: Cigarette smoking prevalence in US counties: 1996-2012
Source: Popul Health Metr. 2014 Mar 24;12:5. doi: 10.1186/1478-7954-12-5 (PMC3987818; doi:10.1186/1478-7954-12-5)
Supplement: Additional file 1 — Detailed description of small area models and data sources. [file 1478-7954-12-5-S1.pdf]

Additional File 1: Cigarette smoking prevalence in US counties: 1996-2012

Small area models

We consider four families of logistic regression models for estimating smoking prevalence in each county. The first family, which we call the 'naïve' model, contains only an intercept, demographic characteristics, a linear time trend, and county-level random slopes and intercepts:

$$Y_{i,k,t} = \text{Binomial}(N_{i,k,t}, p_{i,k,t})$$

$$\text{logit}(p_{i,k,t}) = \nu_{i,k,t} = \beta^{(0)} + \beta^{(1)} \cdot t + \beta_k^{(2)} + \gamma_i^{(0)} + \gamma_i^{(1)} \cdot t$$

where i indicates county, k indicates demographic group (e.g., age, race, etc.), and t indicates calendar year. This model borrows strength by using all data to estimate the mean level ($\beta^{(0)}$), the effect of certain demographic characteristics (given by the $\beta_k^{(2)}$ terms), and the temporal trends ($\beta^{(1)}$) while still allowing for county-level variation through inclusion of the random intercept ($\gamma_i^{(0)}$) and slope ($\gamma_i^{(1)}$).

The second model family, the 'covariate' model, includes everything in the naïve model as well as a series of county-level covariates:

$$\text{logit}(p_{i,k,t}) = \nu_{i,k,t} = \beta^{(0)} + \beta^{(1)} \cdot t + \beta_k^{(2)} + \beta^{(3)} \cdot X_{i,t} + \gamma_i^{(0)} + \gamma_i^{(1)} \cdot t$$

where $X_{i,t}$ is a matrix of county- and state-level covariates and $\beta^{(3)}$ is a vector of regression coefficients corresponding to these covariates. This model borrows strength from external data, making use of variables available at the county level which are related to smoking prevalence. We selected covariates for our model from among those available by performing an exhaustive search: we fit logistic regression models with all combinations of all available covariates and selected the best model based on the Akaike information criterion (AIC) [1]. For smoking prevalence, the covariates we selected were proportion of the county population that is black, proportion of the county population that is American Indian or Alaska native, proportion of the county population that is Hispanic, the proportion of the county

population that holds a bachelor's degree, the proportion of the county population in poverty, the proportion of the county population that is rural, the county-level number of doctors per capita, the county-level unemployment rate, and the state-level cigarette sales per capita. For daily smoking prevalence the same variables were selected except for unemployment. Details of sources for these variables are available in table 1.

The third model family, the 'geospatial' model, includes everything in the naïve model as well as an additional geospatial term which captures spatial information present in the value of the county-level random effects from the naïve model:

$$\mathrm{logit}(p_{i,k,t}) = \nu_{i,k,t} = \beta^{(0)} + \beta^{(1)} \cdot t + \beta_k^{(2)} + \beta^{(4)} \cdot \overline{\delta_\imath} + \gamma_i^{(0)} + \gamma_i^{(1)} \cdot t$$

where for each county $\overline{\delta_\imath}$ is the mean of the estimated $\gamma_i^{(0)}$ for all neighbors (defined by adjacency) from the naïve model. This model borrows strength spatially: we expect that smoking prevalence varies somewhat smoothly in space, so for each county the smoking prevalence of the neighbors is also informative.

The final model family, the 'full' model, includes everything in the previous three models:

$$\mathrm{logit}(p_{i,k,t}) = \nu_{i,k,t} = \beta^{(0)} + \beta^{(1)} \cdot t + \beta_k^{(2)} + \beta^{(3)} \cdot X_{i,t} + \beta^{(4)} \cdot \overline{\delta_\imath} + \gamma_i^{(0)} + \gamma_i^{(1)} \cdot t$$

where all variables are defined as above, except that $\overline{\delta_\imath}$ is calculated based on $\gamma_i^{(0)}$ from the covariate model.

Because we are considering an extended time-period (17 years, from 1996 to 2012), we do not expect that the time trends will be linear over the entire period or that the effect of covariates will necessarily be the same over the entire period. We therefore fit the models using a 'moving window' approach: each model is fit multiple times, using all data in successive, overlapping windows 5 years in length (i.e.

1996-2001, 1997-2002, …, 2008-2012). We then predict for each year using the model centered on that year except for the first two years (1996 and 1997) which use the model fit to the earliest data (1996-2000). In addition to the models fit on 5-year windows, two additional models are fit to just the data from 2011 and 2012 for the purposes of calculating a correction for the omission of cell phones in earlier years, as described in the main text: one that includes all respondents, and one that includes only respondents who can be reached on a landline phone.

We include age in all models as one of the demographic characteristics. Age is grouped into 12 bins: 18-24 years, and then 5-year bins from ages 25 to 74 (i.e. 25-29, 30-34, …, 70-74), and a final bin containing all respondents age 75 and over. We considered inclusion of three other sets of demographic characteristics: race/ethnicity (white non-Hispanic, black non-Hispanic, Hispanic, American Indian or Alaska native, and other), marital status (currently married, formerly married, and never married), and educational attainment (less than high school, high school grad, some college, and college grad). In all four cases, these variables were introduced into the model as a series of indicator covariates where one reference group was absorbed into the overall intercept (age 18-24, white non-Hispanic, formerly married, and less than HS served as the reference groups). Using the validation methods described in the main text, we tested all four model families with all combinations of including or excluding these three sets of demographic characteristics (race, marital status, and education). The models that included education noticeably outperformed the models that excluded education; models that included race and marital status slightly outperformed models that excluded these variables. We therefore considered only models that included all three sets of demographic characteristics. In addition to these demographic characteristics, models were stratified (fit separately) by sex as smoking patterns are known to differ between males and females.

Based on the fitted values of all parameters we are able to generate predictions for every county, sex, age, race, marital status, educational attainment group in each year. We collapse these estimates to county, sex, and age, by year, by finding the weighted mean of the predictions using the county's population by race, marital status, and educational attainment as the weights (see table 1 for details on the source of these populations). Because county-level populations stratified by all these variables simultaneously are not available, we assume that within a given county, sex, and age group for a given year the distributions of the population by race, by marital status, and by educational attainment are independent of each other. Once we have collapsed the estimates to county, sex, age by year, we age-standardize the estimates using the 2000 census population. State and national estimates for each year are derived by population weighting the county-level estimates in the corresponding year. Similarly, estimates for both sexes combined are a weighted average of the male and female estimates using the observed distribution of the adult population by sex in the 2000 census.

The small area models employed require that we have data from each respondent in the BRFSS on their demographic characteristics (i.e. age, sex, race, marital status, and educational attainment), their county of residence, and their smoking status. Table 2 gives information on the total number of respondents and the number of respondents with complete data in each year of BRFSS data and Additional file 2 gives the number of respondents with complete data available in each county for each year. We perform all analyses on respondents who have complete data on all of the variables listed above.

Table 1: Data sources

Use	Source	Notes
**County changes**		
Determining consistent county units of analysis.	Census Bureau[2]	
**County adjacencies**		
Determining neighborhood structure for use in geospatial and full models.	Census Bureau[3]	
**Proportion Black, Hispanic, American Indian or Alaska native, and Asian (county-level)**		
Covariate in covariate and full models.	NCHS Bridged Race Files[4-6]	
**Proportion with a college degree (county-level)**		
Covariate in covariate and full models.	1990 Census[7], 2000 Census[8], 2009-2012 American Community Survey (ACS) 5-yr estimates[9-12]	County-level data are available for 1990, 2000, and 2007-2010. Linear interpolation is used to fill in missing years from 1990 to 2007 and the 2010 values are used for all years after 2010.
**Percent rural (county-level)**		
Covariate in covariate and full models.	1990 Census[13], 2000 Census[14], 2010 Census[15]	Linear interpolation was used to fill in intercensal years. 2010 values are used for all years after 2010.
**Poverty (county-level)**		
Covariate in covariate and full models.	Small Area Income and Poverty Estimates (SAIPE)[16]	County-level data are available for 1989, 1993, 1995, and 1997-2012. Linear interpolation was used to fill in missing years from 1990 to 2012.
**Doctors per capita (county-level)**		
Covariate in covariate and full models.	Area Health Resource File (AHRF)[17]	County-level data are available for 1990, 1995, 2000-2008, 2010, and 2011. Linear interpolation was used to fill in missing years from 1990 to 2011 and 2011 values were used for 2011 and 2012. The variable for 'Non-Federal MDs' was used in place of all MDs as this was available for more years.
**Unemployment (county-level)**		
Covariate in covariate and full models.	Local Area Unemployment Statistics (LAUS)[18]	

**Cigarette sales per capita (state-level)**		
Covariate in covariate and full models.	State Tobacco Activities Tracking & Evaluation System (STATE)[19]	
**County population by age, sex, and race**		
Aggregation of model estimates.	NCHS Bridged Race Files[4-6]	
**County population by age, sex, and marital status**		
Aggregation of model estimates.	2000 Census[20], 2009-2012 American Community Survey (ACS) 5-yr estimates[21-24]	County-level data are available from the census in 2000 and from the 5-year ACS estimates published in 2009-2012, corresponding to estimates in 2007-2010. We use linear interpolation to fill in years between 2000 and 2007 and we use the value in 2000 for all years before 2000 and the value in 2010 for all years after 2010.
**County population by age, sex, and educational attainment**		
Aggregation of model estimates.	2000 Census[25], 2009-2012 American Community Survey (ACS) 5-yr estimates[26-29]	County-level data are available from the census in 2000 and from the 5-year ACS estimates published in 2009-2012, corresponding to estimates in 2007-2010. We use linear interpolation to fill in years between 2000 and 2007 and we use the value in 2000 for all years before 2000 and the value in 2010 for all years after 2010.
**Phone usage patterns**		
Aggregation of model estimates in 2011-2012.	Blumberg et al.[30]	Data are available for 2011 only, so the 2011 values are applied to 2011 and 2012. Estimates are available for 93 non-overlapping geographic areas consisting of states, counties, or groups of counties. We apply the estimate for each state, county, or group of counties to all counties in the aggregate.
**Age and sex standard**		
Age standardizing model estimates and combining male and female estimates.	2000 Census[31]	

County and state shape files		
Creating maps.	SEER*Stat Bridge[32]	

Table 2: BRFSS Data

Survey Year	Total Respondents	Missing Age	Missing Race	Missing Education	Missing Marital Status	Missing County	Missing Smoking Status	Total Respondents Included in Analysis	Number Counties Represented
1996	122,268	506 (0.4%)	425 (0.3%)	322 (0.3%)	305 (0.2%)	1,652 (1.4%)	318 (0.3%)	119,154	2,908
1997	133,321	697 (0.5%)	602 (0.5%)	342 (0.3%)	359 (0.3%)	1,324 (1.0%)	348 (0.3%)	130,157	2,951
1998	146,992	656 (0.4%)	707 (0.5%)	409 (0.3%)	393 (0.3%)	2,104 (1.4%)	378 (0.3%)	143,055	3,068
1999	156,937	842 (0.5%)	786 (0.5%)	445 (0.3%)	408 (0.3%)	1,671 (1.1%)	451 (0.3%)	153,077	3,071
2000	180,244	1,105 (0.6%)	1,152 (0.6%)	464 (0.3%)	570 (0.3%)	2,245 (1.2%)	519 (0.3%)	175,014	3,089
2001	205,140	2,119 (1.0%)	2,197 (1.1%)	594 (0.3%)	783 (0.4%)	4,043 (2.0%)	645 (0.3%)	196,163	3,109
2002	240,735	1,883 (0.8%)	2,450 (1.0%)	542 (0.2%)	766 (0.3%)	3,726 (1.5%)	685 (0.3%)	231,936	3,106
2003	257,659	2,002 (0.8%)	2,208 (0.9%)	605 (0.2%)	832 (0.3%)	3,336 (1.3%)	693 (0.3%)	249,194	3,101
2004	299,443	1,977 (0.7%)	2,919 (1.0%)	736 (0.2%)	1,088 (0.4%)	3,868 (1.3%)	990 (0.3%)	289,367	3,106
2005	352,843	2,654 (0.8%)	3,398 (1.0%)	876 (0.2%)	1,307 (0.4%)	4,976 (1.4%)	1,525 (0.4%)	339,974	3,103
2006	349,924	3,339 (1.0%)	3,757 (1.1%)	966 (0.3%)	1,497 (0.4%)	15,942 (4.6%)	1,463 (0.4%)	325,512	2,808
2007	426,347	3,598 (0.8%)	4,211 (1.0%)	1,229 (0.3%)	1,624 (0.4%)	22,815 (5.4%)	1,792 (0.4%)	393,931	2,812
2008	409,031	3,586 (0.9%)	4,270 (1.0%)	1,237 (0.3%)	1,651 (0.4%)	28,805 (7.0%)	1,632 (0.4%)	370,996	2,406
2009	426,925	3,653 (0.9%)	4,737 (1.1%)	1,480 (0.3%)	1,872 (0.4%)	35,303 (8.3%)	2,751 (0.6%)	381,002	2,283
2010	446,200	4,160 (0.9%)	6,256 (1.4%)	1,578 (0.4%)	2,120 (0.5%)	38,949 (8.7%)	2,909 (0.7%)	394,757	2,278
2011	500,550	4,950 (1.0%)	6,102 (1.2%)	1,925 (0.4%)	2,629 (0.5%)	47,842 (9.6%)	2,546 (0.5%)	438,170	2,274
2012	471,340	4,579 (1.0%)	6,279 (1.3%)	1,913 (0.4%)	2,864 (0.6%)	46,406 (9.8%)	9,612 (2.0%)	406,797	2,277
**All**	**5,125,899**	**42,306 (0.8%)**	**52,456 (1.0%)**	**15,663 (0.3%)**	**21,068 (0.4%)**	**265,007 (5.2%)**	**29,257 (0.6%)**	**4,738,256**	**3,127**

References

[1] Akaike H. A new look at the statistical model identification. IEEE Transactions on Automatic Control. 1974;19(6):716–23.

[2] US Census Bureau. Substantial Changes to Counties and County Equivalent Entities: 1970-Present. Available from: http://www.census.gov/geo/reference/county-changes.html.

[3] US Census Bureau. United States County Adjacency 2010. Available from: http://www.census.gov/geo/reference/county-adjacency.html.

[4] National Center for Health Statistics (NCHS), Centers for Disease Control and Prevention (CDC), US Census Bureau. United States Bridged-Race Intercensal Population Estimates 1990-1999. Hyattsville, United States: National Center for Health Statistics (NCHS), Centers for Disease Control and Prevention (CDC), 2004. Available from: http://www.cdc.gov/nchs/nvss/bridged_race/data_documentation.htm#july1999.

[5] National Center for Health Statistics (NCHS), Centers for Disease Control and Prevention (CDC), US Census Bureau. United States Bridged-Race Intercensal Population Estimates 2000-2009. Hyattsville, United States: National Center for Health Statistics (NCHS), Centers for Disease Control and Prevention (CDC), 2012. Available from: http://www.cdc.gov/nchs/nvss/bridged_race/data_documentation.htm#july2009.

[6] National Center for Health Statistics (NCHS), Centers for Disease Control and Prevention (CDC), US Census Bureau. United States Vintage 2012 Bridged-Race Postcensal Population Estimates 2010-2012. Hyattsville, United States: National Center for Health Statistics (NCHS), Centers for Disease Control and Prevention (CDC), 2013. Available from: http://www.cdc.gov/nchs/nvss/bridged_race/data_documentation.htm#vintage2012.

[7] US Census Bureau. 1990 US Census, Summary Tape File 3 (STF3), Table P057: Educational Attainment. Available from: http://www2.census.gov/census_1990/1990STF3.html.

[8] US Census Bureau. 2000 US Census, Summary File 3 (SF3), Table DP-2: Profile of Selected Social Characteristics. Generated using American FactFinder: http://factfinder2.census.gov.

[9] US Census Bureau. 2009 American Community Survey 5-year Estimates, Table S1501: Educational Attainment. Generated using American FactFinder: http://factfinder2.census.gov.

[10] US Census Bureau. 2010 American Community Survey 5-year Estimates, Table S1501: Educational Attainment. Generated using American FactFinder: http://factfinder2.census.gov.

[11] US Census Bureau. 2011 American Community Survey 5-year Estimates, Table S1501: Educational Attainment. Generated using American FactFinder: http://factfinder2.census.gov.

[12] US Census Bureau. 2012 American Community Survey 5-year Estimates, Table S1501: Educational Attainment. Generated using American FactFinder: http://factfinder2.census.gov.

[13] US Census Bureau. 1990 US Census, Summary Tape File 1 (STF1), Table H004: Urban and Rural. Available from: http://www2.census.gov/census_1990/1990STF1.html.

[14] US Census Bureau. 2000 US Census, Summary File 1 (SF1), Table H002: Urban and Rural. Generated using American FactFinder: http://factfinder2.census.gov.

[15] US Census Bureau. 2010 US Census, Summary File 1 (SF1), Table H2: Urban and Rural. Generated using American FactFinder: http://factfinder2.census.gov.

[16] US Census Bureau. United States Small Area Income and Poverty Estimates 1989, 1993, 1995, 1997-2012. Washington, DC, United States: US Census Bureau, 2013. Available from: http://www.census.gov/did/www/saipe/data/index.html.

[17] US Department of Health and Human Services, Health Resources and Services Administration. Area Health Resources File 2012-2013. Washington, DC, United States: US Department of Health and Human Services, Health Resource and Services Administration, 2013. Available from: http://arf.hrsa.gov/download.htm.

[18] US Bureau of Labor Statistics. Local Area Unemployment Statistics. 2013. Available from: ftp://ftp.bls.gov/pub/time.series/la/.

[19] Centers for Disease Control and Prevention (CDC). State Tobacco Activities Tracking and Evaluation System, Economics, Cigarette Sales. 2013. Available from: http://apps.nccd.cdc.gov/statesystem/TrendReport/TrendReports.aspx.

[20] US Census Bureau. 2000 US Census, Summary File 3 (SF3), Table PCT007: Sex by Marital Status by Age for the Population 15 Years and Over. Generated using American FactFinder: http://factfinder2.census.gov.

[21] US Census Bureau. 2009 American Community Survey 5-year Estimates, Table B12002: Sex by Marital Status by Age for the Population 15 Years and Over. Generated using American FactFinder: http://factfinder2.census.gov.

[22] US Census Bureau. 2010 American Community Survey 5-year Estimates, Table B12002: Sex by Marital Status by Age for the Population 15 Years and Over. Generated using American FactFinder: http://factfinder2.census.gov.

[23] US Census Bureau. 2011 American Community Survey 5-year Estimates, Table B12002: Sex by Marital Status by Age for the Population 15 Years and Over. Generated using American FactFinder: http://factfinder2.census.gov.

[24] US Census Bureau. 2012 American Community Survey 5-year Estimates, Table B12002: Sex by Marital Status by Age for the Population 15 Years and Over. Generated using American FactFinder: http://factfinder2.census.gov.

[25] US Census Bureau. 2000 US Census, Summary File 3 (SF3), Table PCT025: Sex by Age by Educational Attainment for the Population 18 Years and Over. Generated using American FactFinder: http://factfinder2.census.gov.

[26] US Census Bureau. 2009 American Community Survey 5-year Estimates, Table B15001: Sex by Age by Educational Attainment for the Population 18 Years and Over. Generated using American FactFinder: http://factfinder2.census.gov.

[27] US Census Bureau. 2010 American Community Survey 5-year Estimates, Table B15001: Sex by Age by Educational Attainment for the Population 18 Years and Over. Generated using American FactFinder: http://factfinder2.census.gov.

[28] US Census Bureau. 2011 American Community Survey 5-year Estimates, Table B15001: Sex by Age by Educational Attainment for the Population 18 Years and Over. Generated using American FactFinder: http://factfinder2.census.gov.

[29] US Census Bureau. 2012 American Community Survey 5-year Estimates, Table B15001: Sex by Age by Educational Attainment for the Population 18 Years and Over. Generated using American FactFinder: http://factfinder2.census.gov.

[30] Blumberg SJ, Luke JV, Ganesh, N, Davern ME, Boudreaux MH. Wireless Substitution: State-level Estimates From the National Health Interview Survey, 2010-2011. National Health Statistics Reports. 2012; 61. Available from: http://www.cdc.gov/nchs/data/nhsr/nhsr061.pdf.

[31] US Census Bureau. 2000 US Census, Summary File 1 (SF1), Table QTP1: Age Groups and Sex. Generated using American FactFinder: http://factfinder2.census.gov.

[32] National Cancer Institute. SEER Stat Bridge State and County FIPS Codes 2000-2004. Available from: http://gis.cancer.gov/tools/seerstat_bridge/fips_vars/#sc_2000_2004.
